# Efficacy of Painting Wind Turbine Blades as a Mitigation Measure to Reduce Bird Collisions in a Mediterranean Wind Farm

**DOI:** 10.1002/ece3.74017

**Published:** 2026-09-14

**Authors:** Roel May, Miguel Ángel Hernández Gómez, Juan Luis Aguirre Martínez, Jenifer Andreu Miguel

**Affiliations:** ^1^ Norwegian Institute for Nature Research (NINA) Trondheim Norway; ^2^ Department of Life Sciences University of Alcalá Madrid Spain; ^3^ ACCIONA Energía Madrid Spain

**Keywords:** birds of prey, blade patterning, collision fatalities, El Ruedo wind farm, mitigation, passerines

## Abstract

One of the most notable impacts of wind energy is bird mortality due to collisions with wind turbines, making it necessary to adopt measures to prevent or mitigate this impact. In recent years, studies have been carried out to test the efficacy of painting wind turbine blades to increase their visibility and forewarn birds, thus preventing collisions. To corroborate these studies in other site‐ and species‐specific settings, we report on a long‐term experiment (2012–2024) performed at the El Ruedo wind farm in Cadiz, Spain. One of three rotor blades was painted black at four of the 20 wind turbines, underneath which daily fatality searches were performed both before (9.5 years) and after (3.5 years) treatment. This Before–After–Control–Impact experiment resulted in a significant reduction in bird fatality rates of 74% at the painted turbines relative to unpainted control turbines. The treatment had the largest effect in reducing passerine fatalities as well as large waterbirds, but less so for birds of prey. To which extent local abundance, flight activity or visual fields affect the measure's efficacy remains as yet unclear. As this mitigation measure has now been shown to be functional at sites spanning a wide latitudinal range, it is paramount to overcome implementation hurdles and evaluate other site‐ and species‐specific conditions.

## Graphical abstract

Bird mortality due to collisions with wind turbines necessitates the implementation of measures to mitigate this impact. Blade patterning has shown to be a promising measure to increase turbine visibility to birds and reduce collisions. Employing a Before–After–Control–Impact experiment using long‐term fatality searches, bird fatality rates were reduced by 74% at the painted turbines relative to unpainted control turbines; corroborating earlier studies in another site‐ and species‐specific setting.

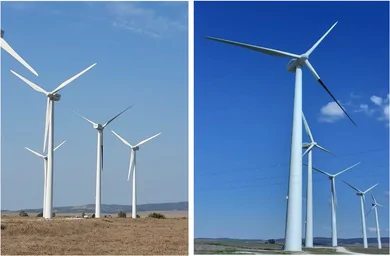

## 1 Introduction

Climate change concerns (UNFCCC 2016) have boosted the innovation, development and application of renewable energy sources worldwide (IEA 2021; IPCC 2022; IRENA 2019). Given its considerable potential (Duffy et al. 2020; Ryberg et al. 2019), wind energy is regarded as an important renewable energy source to secure national energy supply and adhere to international calls and agreements to reduce greenhouse gas emissions. However, it can also have strong negative effects on biodiversity through habitat loss, disturbance, collision and barrier effects with the likelihood and magnitude of those being site‐ and species‐specific (Katzner et al. 2020; Katzner et al. 2025).

Bird and bat fatalities of birds and bats from wind turbine collisions have raised the most ecological concern, even though wind turbine‐induced mortality is often lower than that caused by other human infrastructure (Loss et al. 2015). Collisions with wind turbines do not affect all groups of birds equally. Diurnal birds of prey and migratory birds are thought to have the highest risk of collision (Morant et al. 2025; Thaxter et al. 2017). To which extent fatality estimates, relative to a species' abundance, are biased towards larger‐bodied species as an artefact of their greater detectability remains however unknown (AWWI 2021; de Lucas et al. 2008). For instance, passerines often account for the majority of bird mortality due to collisions with wind turbines, but they are also the most numerous of terrestrial birds (AWWI 2021; Morant et al. 2025) and may be detected less (Nilsson et al. 2023). Still, the species known to be susceptible do possess characteristics that enhance collision risk. The high wing loading in soaring birds, including migratory species such as vultures, cranes and storks, may optimise soaring but makes them less manoeuvrable in the absence of adequate air currents (Santos et al. 2017; Santos et al. 2020; Tucker 1971). Areas with thermal updrafts in warmer climates and orographic updrafts, usually found along ridges and steep slopes, coincide with good sites for wind turbines (Hanssen et al. 2020; Katzner et al. 2012; Marques et al. 2019). Scavenging species, such as vultures and kites, scan the ground for carrion while flying, which, when combined with their reduced sagittal visual field (Martin et al. 2012; Martin and Shaw 2010), creates a blind spot in front of them hindering the timely detection of obstacles in front of them. Soaring birds that migrate in large flocks, such as vultures, kites and storks, make them particularly vulnerable to interactions with wind turbines when crossing migration bottlenecks (Assandri et al. 2024; Gauld et al. 2022). Finally, the longevity and low recruitment rates of birds of prey make that collision mortality has a relatively large impact at the population level (Beston et al. 2016; Carrete et al. 2009; May et al. 2019).

Developing effective and practical measures to reduce such impacts is therefore important (May 2019). In practice, knowledge on the efficacy of mitigation measures is however limited as the route from documenting an impact to successful mitigation is often very long (Marshall 2001) and requires collaborative action to test and document its efficacy (Gottlieb et al. 2025; May 2023). Various methods have been proposed, implemented and tested to prevent and reduce bird mortality at wind farms (May et al. 2015). These include curtailment of wind turbines (Ferrer et al. 2022; McClure et al. 2022) and acoustic deterrence using radar‐ or camera‐based detection systems (Dorey et al. 2019; Smith et al. 2025). While such systems have been proven to be cost‐effective for bats (Whitby et al. 2024), this is less the case for birds (Garcia‐Rosa and Tande 2023; Smallwood and Bell 2020). The past years, blade patterning has gained international interest as a cost‐effective mitigation measure to reduce bird collisions. Laboratory experiments with American kestrels ( *Falco sparverius* ) suggested that painting one of the rotor blades black can improve its detectability by reducing morion smear (Hodos 2003). These experiments triggered the in situ study to verify the effectiveness of this mitigation measure at the Smøla wind farm in Norway, concluding that it can reduce bird fatality rates by up to 70% (May et al. 2020). However, they stressed that these results should not be extrapolated to other cases and recommend conducting further studies to verify whether this measure has similar effects under other circumstances (e.g., geographical features, climate conditions, species composition). Recently, a follow‐up study carried out in the Netherlands did not result in a significant reduction of fatality rates (Kleyheeg‐Hartman et al. 2025). Another study in South‐Africa found a decrease of 83% in fatality rates, relative to control turbines, after painting one‐of‐three rotor blades red instead of black (Simmons et al. 2026). With the aim of strengthening the knowledge base on the level of efficacy of blade patterning on bird fatality rates, a study was carried out in southern Spain to verify the results of this mitigation measure within another site‐ and species‐specific context.

## 2 Material and Methods

### 2.1 Study Area and Experimental Set‐Up

**FIGURE 1:**
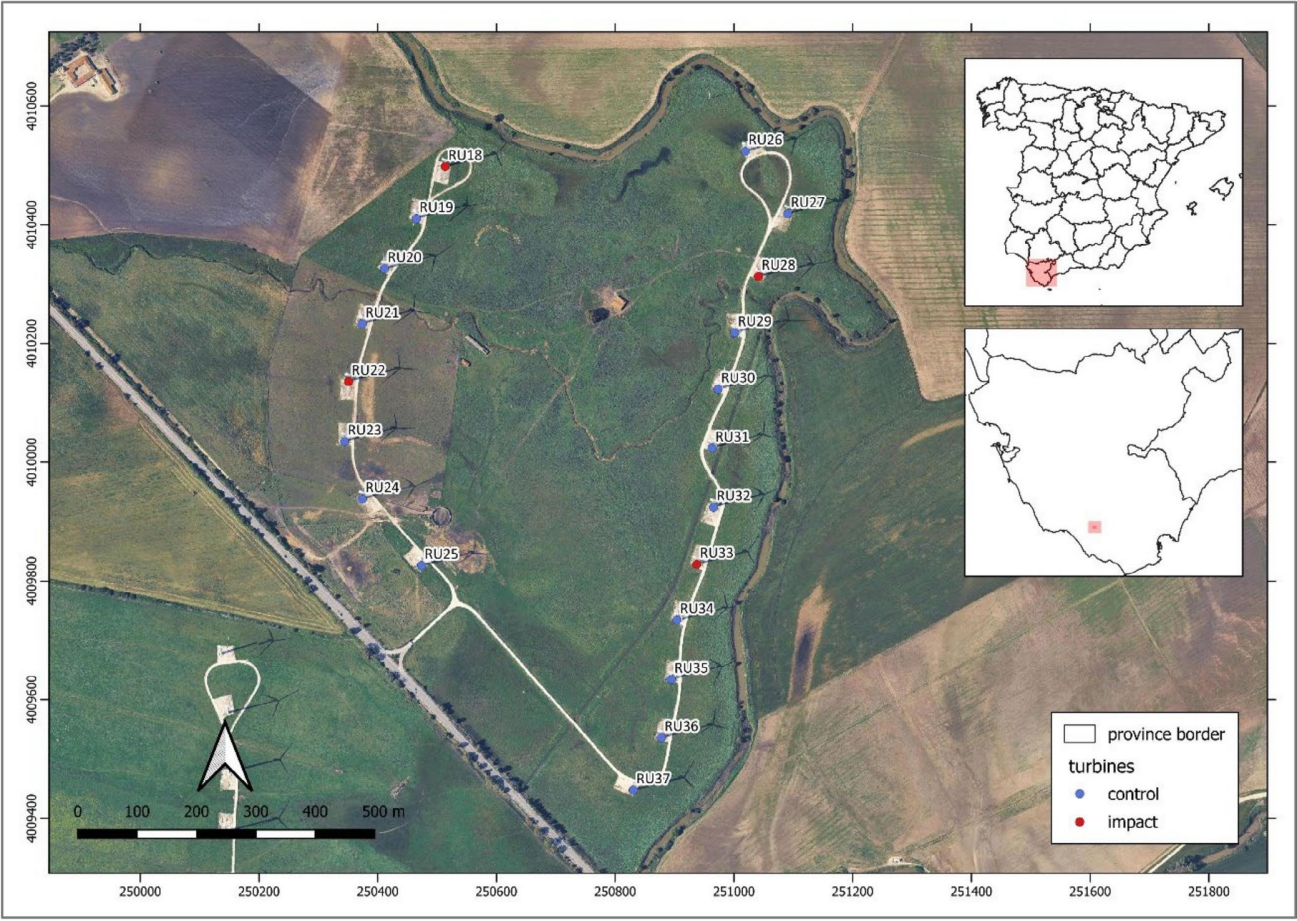
Set‐up of the experimental design at the El Ruedo wind farm, Spain. Numbered circles indicate the wind turbines. Four turbines had painted rotor blades (red) while the other turbines functioned as controls (blue).

The wind farm “El Ruedo” is located in the municipality of Tarifa, in Campo de Gibraltar, region of Cádiz, Spain (Figure 1). The wind farm has a capacity of 16 MW and 20 wind turbines with a power of 800 kW each. It is situated in the Almodóvar River plane, at sea level, and flanked by small mountain ranges to the northwest (Sierras del Retín) and southeast (Sierras de la Plata). The vegetation in this area consists of a mosaic landscape comprised of arid vegetation and agricultural fields, with the wind farm situated within a patch of arid grassland. This grassland vegetation consists of low‐growing herbaceous therophytes, geophytes and perennial hemicryptophytes typical of dry Mediterranean grasslands; dominated by nitrophilous species as a result of intensive bovine livestock use. It has low to medium ground cover with significant areas of bare soil. The low to medium ground coverage and the low‐growing species facilitate the detectability of fatalities. Regionally, it lies in the vicinity of the Strait of Gibraltar, which forms the shortest Mediterranean Sea crossing between the Iberian Peninsula and North Africa. This area is therefore of great ornithological value, especially for migratory birds as a major flyway between Eurasia and Africa, since its geographical location funnels the passage of millions of individuals during Spring and Autumn migration (Lathbury 1970; Newton 2007).

**FIGURE 2:**
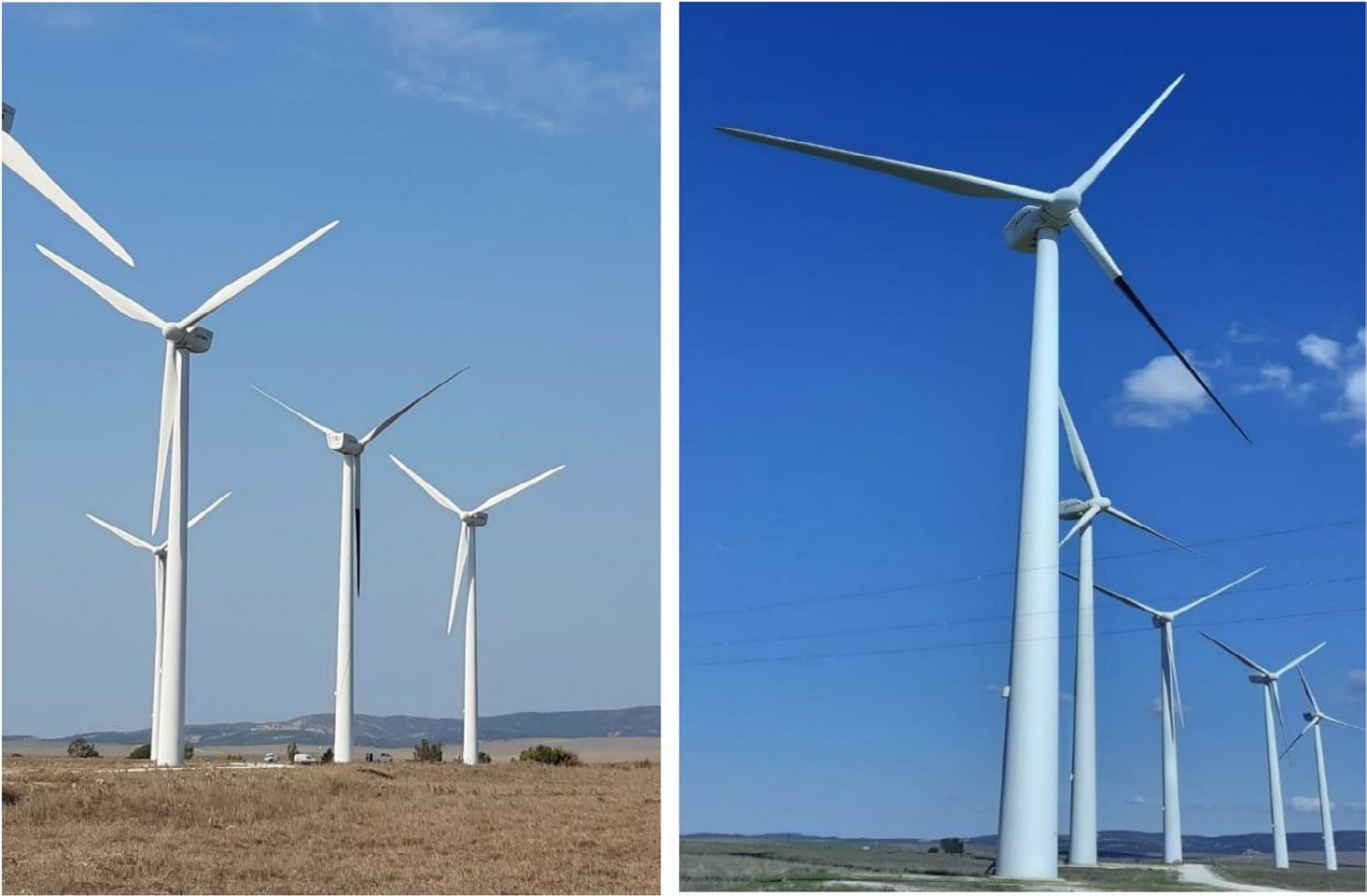
Wind turbines at the El Ruedo wind farm with a blade painted black as a visual cue to birds to reduce collision fatalities.

To assess the effect of the patterned blades on fatality rates, one of the three rotor blades was painted black at four wind turbines spread within the wind farm (Figure 2). The blades of the following wind turbines were painted on the dates indicated: RU‐18 (01.12.2020), RU‐22 (29.09.2021), RU‐28 (28.09.2021), and RU‐33 (05.10.2021) (Figure 1). The remaining 16 wind turbines were used in the analyses as controls, to compare the results between wind turbines where the measure was applied and those where it was not (i.e., Before‐After‐Control‐Impact design).

### 2.2 Fatality Search Regime

Searches for bird fatalities were performed on a daily basis between 02.01.2012 and 31.12.2024; rendering 9.5 years before and 3.5 years after treatment. A minimum area of 32.5 m around the wind turbine (3318 m^2^, 10% larger than the rotor diameter) was inspected on foot by at least one person. To do so, parallel linear transects were carried out, separated by no more than 5 m. When wind turbines were in areas with cattle, the inspection was performed from elevated vantage points using binoculars, conducting a detailed survey in search of carcasses, feathers, or other remains or signs indicating the presence of injured or deceased individuals.

Although no observer detection and carcass removal trials were performed, the daily monitoring in open grassy vegetation is assumed to limit any biases in the fatality data. However, since a comparative analysis is carried out between the painted wind turbines and the control wind turbines within the same wind farm with the same monitoring regime for all wind turbines, the results obtained from the analysis will still be suitable for determining the (relative) efficacy of blade patterning.

### 2.3 Statistical Analysis

Effects of rotor blade patterning on annual fatality rates were tested following a Before–After–Control–Impact (BACI) approach (cf. May et al. 2020). The analyses were performed based on aggregating the number of fatalities per turbine and by year (2012–2024). As the rotor blades were painted on different dates, the before‐after segmentation at control wind turbines was set at the mean of painting dates (16‐07‐2021).

This annual number of fatalities was used as response variable while including the logarithm of the search effort as an offset term (rendering annual fatality rates per turbine). The model included the fixed categorical effects before and after painting (BA), control and painted (impact) turbines (CI) as well as their interaction term (BA:CI). The latter evaluates changes at the impact turbines before and after painting relative to control turbines, to which we refer to in the remainder of the text. To control for any potential effects of turbine IDs and year, random effects were included using a generalised linear mixed‐effects model with a Poisson distribution using the glmer function of the lme4 library (Bates et al. 2015) in the statistical software program R 4.5.0 (R Core Team 2025). To control for potential over‐dispersion in the data we also included an observation‐level random effect (Harrison 2014). A similar model was constructed by aggregating across seasons instead of year to enable the assessment of seasonal effects. Seasons were defined using equal Julian day intervals (spring: 75–166; summer: 167–258; autumn: 259–349; winter: 350–74). These models were constructed for all recorded bird fatalities and for specific species groups (birds of prey (raptor, vulture and owl); passerines; large waterbirds (heron, stork, spoonbill, night heron); aerial insectivores (swift, swallow, martin); other bird species). To test for the potential effect of birds potentially being forced towards neighbouring turbines due to deterrence from painted turbines, we first verified whether these controls (as ‘pseudo‐impact’ turbines) differed significantly in annual fatality rates from other untreated turbines (as unrelated controls) before‐after treatment (thus excluding the impact turbines from this test).

To further assess the overall effects of painting on the bird groups (see Appendix 1), we estimated the (Poisson) probability for obtaining the actual recorded number of fatalities in the period after painting, assuming no treatment effect. We estimated the expected number of fatalities over the total number of searches executed at the painted turbines after painting with an expected mean annual fatality rate assuming no effect of the treatment: ${\text{rate}}_{\mathrm{IA}}=\frac{{\text{rate}}_{\mathrm{CA}}}{{\text{rate}}_{\mathrm{CB}}}\cdot {\text{rate}}_{\mathrm{IB}}$ using the rpois function where the rates indicate the four different groups of the BACI design. The probability was obtained through a simulation with 10,000 iterations to derive the proportion of times we obtained the same number of fatalities as were actually recorded. Also, the expected number of fatalities (mean and S.D.) assuming no treatment effect was calculated. To estimate the likely reduction effect of the treatment, group‐specific generalised mixed effects BACI‐models were run as described above but which were aggregated across years (hence only including turbine ID as random effect).

## 3 Results

In the period 2012–2024, 297 fatalities were recorded during the daily searches, including 44 bird species (Appendix 1). The ten most common species that were found were: corn bunting ( *Emberiza calandra* ) [55], western cattle egret ( *Bubulcus ibis* ) [41], white stork ( *Ciconia ciconia* ) [25], house sparrow ( *Passer domesticus* ) [18], Eurasian griffon vulture ( *Gyps fulvus* ) [18], common kestrel ( *Falco tinnunculus* ) [17], crested lark ( *Galerida cristata* ) [12], common swift ( *Apus apus* ) [11], black kite ( *Milvus migrans* ) [10] and booted eagle ( *Hieraaetus pennatus* ) [10]. On average, 23 ± 9 S.D. bird fatalities were found annually in the wind farm. On a per‐turbine basis, the number of fatalities decreased from 1.77 to 0.29 at the patterned turbines, while the control turbines saw a decrease from 1.11 to 0.69 fatalities per turbine per year.

**FIGURE 3:**
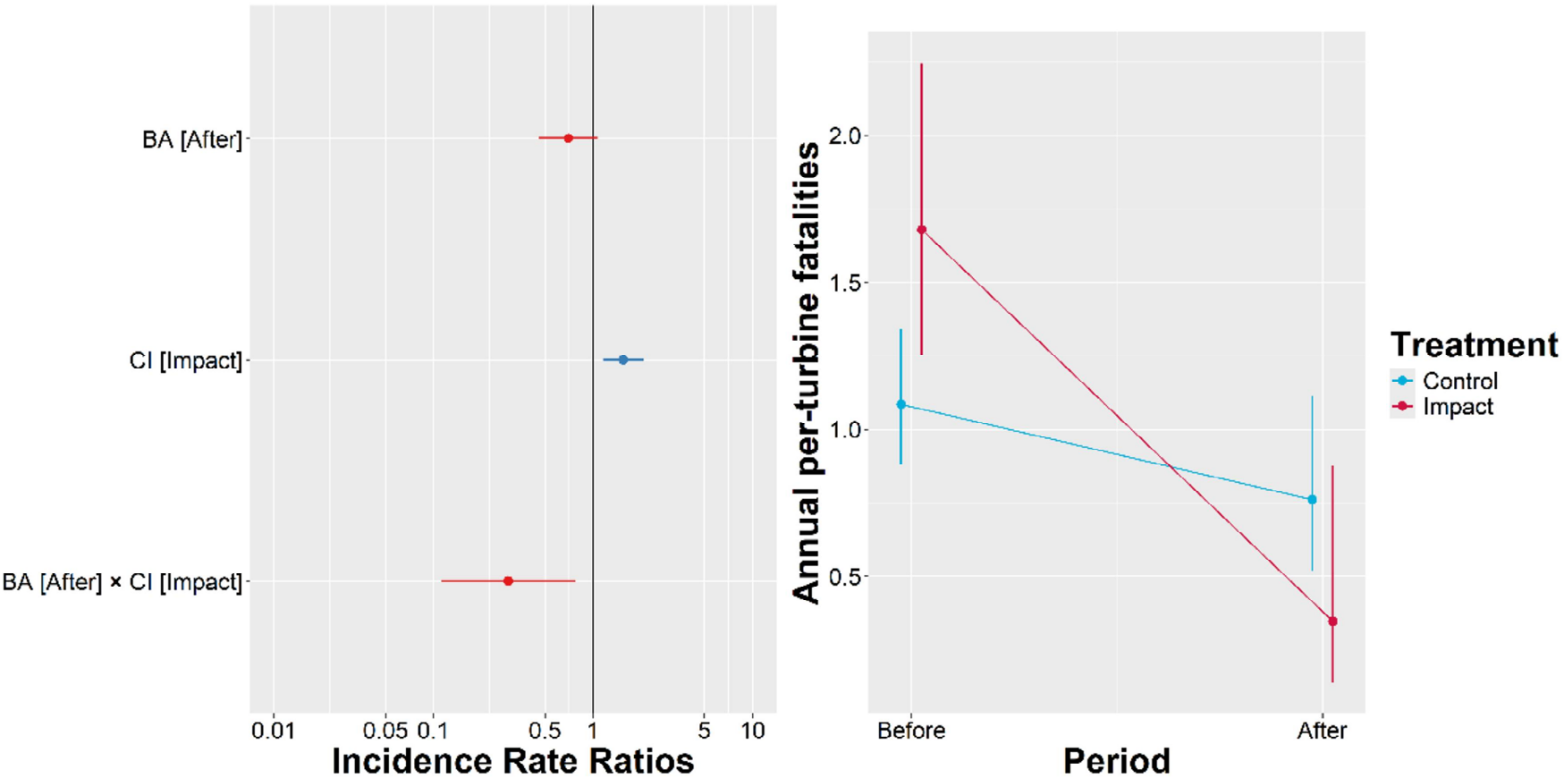
Result of the BACI‐model testing for the effect of contrast painting of rotor blades on the annual fatality rates at the El Ruedo wind farm. BA refers to before vs after treatment and CI to control vs impact (i.e., unpainted vs painted) turbines; BA:CI represents the interaction between time and treatment. Reference states are indicated in brackets. Left: Incidence rate ratios for the fixed categorical effects in the generalised mixed effects model. Positive and negative effects are indicated in blue and red, respectively. Right: Estimated effect of contrast treatment before and after painting at the control (blue) and impact (red) turbines.

**FIGURE 4:**
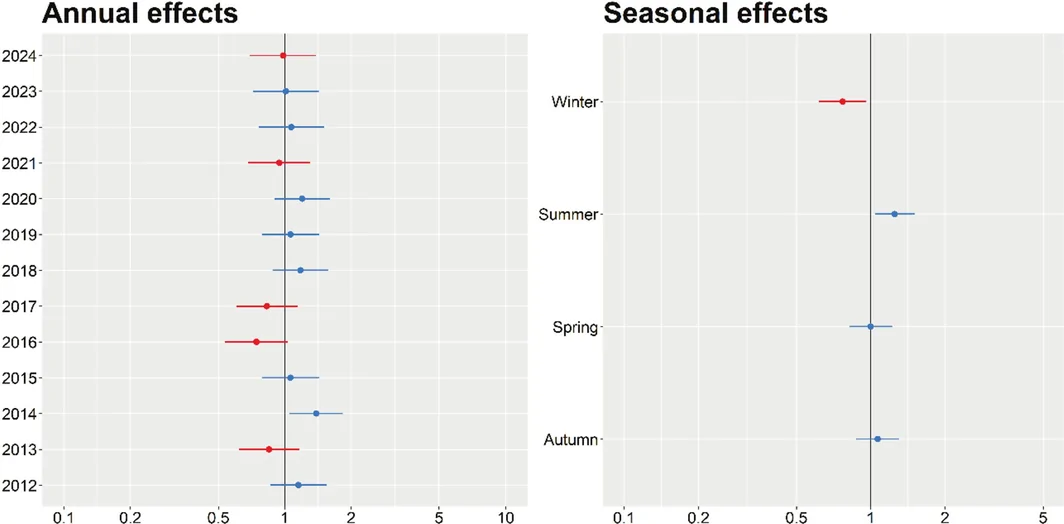
Annual and seasonal variation in annual fatality rates at the El Ruedo wind farm. Positive and negative effects are indicated in blue and red, respectively.

**FIGURE 5:**
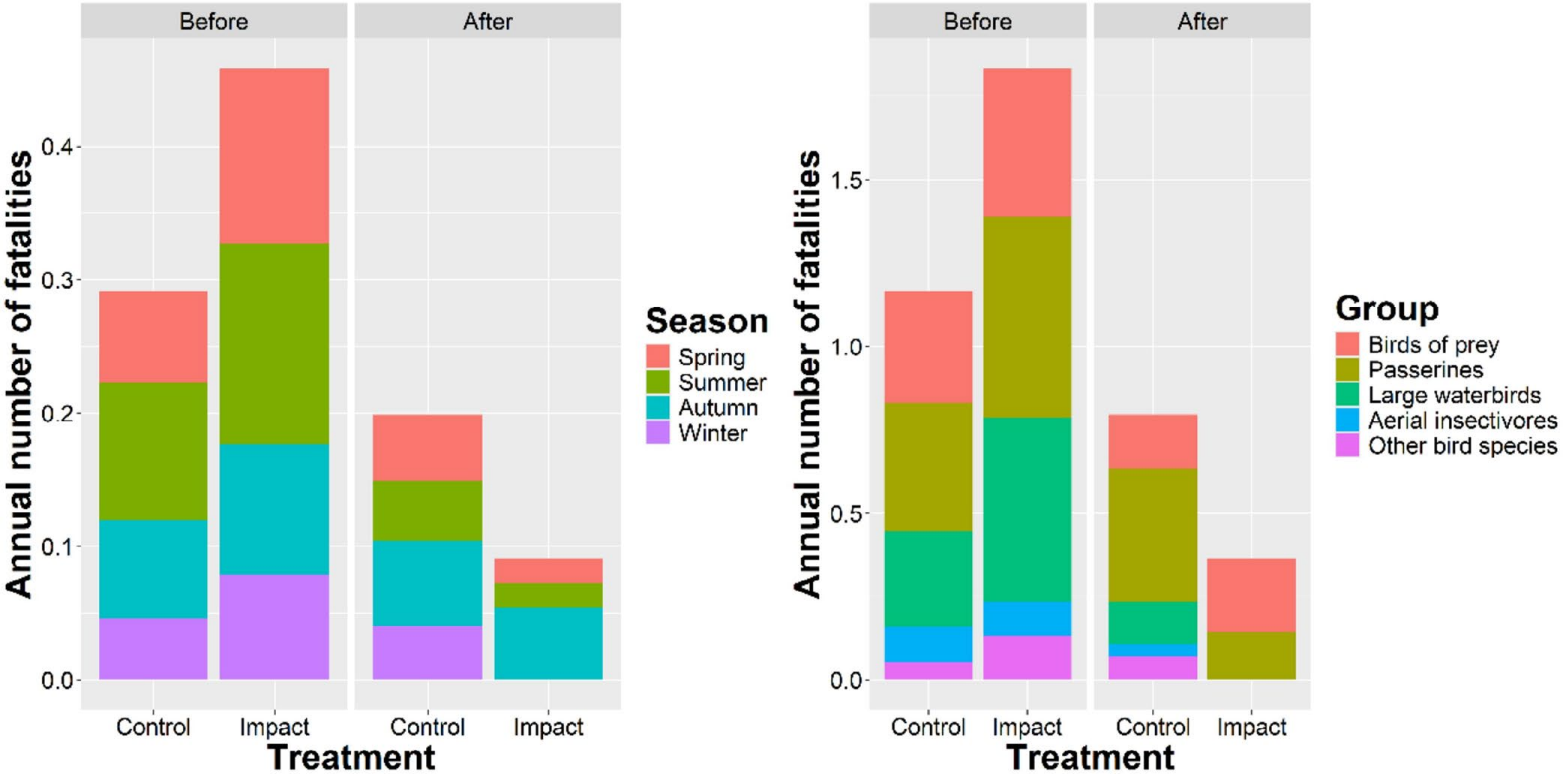
Distribution of annual number of fatalities per turbine across seasons (left panel) and species groups (right panel) at the El Ruedo wind farm. Control and Impact refer to unpainted and painted turbines, respectively. Before and After refer to timing relative to treatment.

Appling statistics, we found no effect of a higher probability of fatalities at turbines neighbouring the painted turbines relative other untreated turbines within the wind farm before‐after treatment (*z* = 1.101, *p* = 0.271, *N* = 224). The annual bird fatality rates were significantly reduced after treatment at the painted turbines (*z* = −2.457, *p* = 0.014, *N* = 280; Table 1 and Figure 3). Overall, there was an average 73.8% reduction in the annual bird fatality rates after painting at the painted turbines relative to the control turbines (95% CI: 73.6%–74.1%). When grouping data by season instead of years, painting reduced seasonal fatality rates by 74.6% (95% CI: 74.3%–74.8%; *z* = −2.491, *p* = 0.013, *N* = 160; Table 1). The annual and seasonal fatality rates fluctuated among years and seasons, respectively (variance Year: 0.051; variance Season: 0.041, Figure 4); stressing the necessity of long‐term studies. Seasonally, fatality rates were –relative to the control turbines– reduced at the painted turbines after treatment strongest during winter (100%), followed by spring (83%) and summer (68%) and least during autumn (33%) (Figure 5).

**TABLE 1:** Model estimates testing the effect of painting on the annual fatality rate of birds found at the El Ruedo wind farm (2012–2024) using a Before–After–Control–Impact (BACI) design. BA refers to before vs after treatment and CI to control vs impact (i.e., unpainted vs painted) turbines; BA:CI represents the interaction between time and treatment. The model controlled for (log‐transformed) search effort using an offset term.

Fixed effects	Annual fatality rates				Seasonal fatality rates			
	Estimate	S.E.	*z*‐value	*p*	Estimate	S.E.	*z*‐value	*p*
Intercept	−5.819	0.114	−51.207	< 0.001	−5.769	0.130	−44.444	< 0.001
BA – After	−0.355	0.217	−1.638	0.101	−0.396	0.169	−2.346	0.019
CI – Impact	0.437	0.150	2.919	0.004	0.449	0.143	3.140	0.002
BA:CI	−1.223	0.498	−2.457	0.014	−1.227	0.492	−2.491	0.013

Random intercepts	Variance	S.D.	*N*	Variance	S.D.	*N*
Record	0.079	0.281	280	0.008	0.091	160
Turbine	0.000	0.000	20	0.000	0.000	20
Season				0.041	0.203	4
Year	0.051	0.226	13			

The (Poisson) probability for recording the actually found fatalities in the period after painting for specific species groups, given a prior mean annual fatality rate, was estimated to lead to a significant reduction in fatalities for passerines and for large waterbirds (Table 2). For passerines, the overall estimated reduction in annual fatality rates was 74% (*p* = 0.008), while for large waterbirds this amounted to 100% (*p* = 0.035) as no fatalities were found at the impact turbines after painting. The simulation test rendered a non‐significant effect (*p* = 0.224) of the treatment on the number of birds of prey fatalities at the impact turbines after painting of 16%. For aerial insectivores, no significant reduction (*p* = 0.601) was found, and for other bird species, a near‐significant reduction (*p* = 0.076).

**TABLE 2:** Statistics of the total number of recorded fatalities (2012–2024), expected (*N*_exp_ ± S.D.) and actual number (*N*_act_) and likelihood (*p*) of fatalities at impact turbines after painting, based on a prior rate assuming no effect. The reduction was estimated by running group‐specific generalised mixed effects BACI‐models aggregated across years.

Species group	Total	*N*_act_	*p*	Prior rate	*N*_exp_	S.D.	Reduction
Birds of prey	80	3	0.224	0.0006	3.002	1.720	0.160 [0.143–0.176]
Passerines	106	2	0.008	0.0017	8.578	2.900	0.736 [0.732–0.741]
Large waterbirds	72	0	0.035	0.0007	3.323	1.822	1^a^
Aerial insectivores	22	0	0.601	0.0001	0.508	0.714	1^a^
Other bird species	17	0	0.076	0.0005	2.535	1.581	1^a^

^a^ Note that these values are caused by the lack of fatalities found at the impact turbines after painting.

## 4 Discussion

The observed decrease in fatality rates by 74% at the El Ruedo wind farm provides further support for the efficacy of blade patterning to reduce bird collisions. It provides a complementary outcome to earlier studies (Kleyheeg‐Hartman et al. 2025; May et al. 2020; Simmons et al. 2026) within yet another set of site‐ and species‐specific settings. As in the study by May et al. (2020), four turbines were painted but all remaining 16 turbines were used as controls. Despite this limited spatial limitation in replicates, the experiment was based on data from a 13‐year time series (2012–2024); rendering 9.5 years before and 3.5 years after treatment. Given the inherent natural variability that affects the effect size both spatially and temporally, it has been stressed to perform long‐term studies to strengthen BACI experiments (Hewitt et al. 2001). Longer time series account for annual variability at the turbines which thus shed light on possible habituation effects to occur. We found no evidence for habituation effects in our study.

Our study found similar results to May et al. (2020) and Simmons et al. (2026) with a reduction in fatality rates by ca. 70%–85% despite the great latitudinal distance between the study sites. The El Ruedo wind farm (consisting of 20 0.8 MW turbines, 4 turbines with black blades) was smaller compared to the two other sites (Smøla wind farm consists of 68 2.0–2.3 MW turbines, 4 turbines with black blades; Umoya wind farm consists of 37 1.8 MW turbines, 4 turbines with red banded blades). Contrary to these outcomes, a study in the Netherlands (Westereems wind farm consists of 50 3.0 MW turbines, 7 turbines with black blades) did not find any significant reductions in fatality rates. In this latter study, the overall fatality rates at the treated turbines reduced somewhat for day‐active species relative to the control turbines, but this was the result of a mixed response as large gull fatalities were reduced while they increased for waders and passerines (Kleyheeg‐Hartman et al. 2025). They speculate that the lack of effect might be due to the large activity of gulls in the area, the short monitoring period (1 year before and 2 years after treatment) and the cluttered industrial surroundings.

In the El Ruedo wind farm, blade patterning had the most effect on passerines, large waterbirds such as herons and storks, while for birds of prey and aerial insectivores no significant decrease in mortality was observed. Despite being one of the groups with the most data, birds of prey did not experience a significant reduction in fatality rates contrary to May et al. (2020) and Simmons et al. (2026). Fatalities of birds of prey were recorded throughout the year, which may well be the combined effect of migratory birds colliding with the wind turbines as well as locally breeding birds. As the latter occur in lower numbers but may frequently use the area for, e.g., foraging, they may well be more at risk compared to temporally abundant but ‘naïve’ migrants (Martín et al. 2018). Although often underestimated (Nilsson et al. 2023), passerines are among the most often recorded wind turbine fatalities (Morant et al. 2025). It is therefore interesting that blade patterning reduced fatalities by 75%. This affected both lower‐flying species such as corn bunting and house sparrow, but also aerial species such as crested skylark as well as common swifts. Long et al. (2011) showed that light‐reflective white surfaces attracted insects, which in turn may increase aerial insectivore activity close to wind turbines. Given these outcomes from daily surveys performed in open vegetation, this shows the importance of intensive search regimes to avoid underestimation due to carcass removal (Domínguez del Valle et al. 2020). Finally, large waterbird fatalities were not recorded at the patterned turbines after treatment. This group, including herons and storks, is known to be susceptible to wind turbine collisions (Thaxter et al. 2017). Both the white stork and western cattle egret, which were recorded most often within this group, are known to spend time within the rotor swept zone (Blas et al. 2020; Wulff et al. 2016). The homogeneously flat terrain with arid open vegetation inside the wind farm will likely enhance thermal convection (Scacco et al. 2019). Soaring birds, including storks, raptors, and vultures, rely on thermal updrafts to gain altitude and are therefore expected to extend their time within the rotor swept zone especially during summer (Gauld et al. 2022; Santos et al. 2017). Overall, the fatality rates at the patterned turbines prior to treatment were higher for all species groups while the rates showed a slight but non‐significant decline over the years. Why this decline occurred is difficult to ascertain: abundances have decreased for passerines (Onrubia and Tellería 2026) but increased for soaring birds (Martín et al. 2016) in the region. Possibly, some patterned turbines might also enhance awareness beyond neighbouring turbines across the wind farm. However, this supposition remains untested.

With the corroboration of earlier studies on the effect blade patterning has in reducing bird fatality rates, overcoming technical and acceptance hurdles that still remain become even more paramount in order to speed up the implementation of functional mitigation measures in practice (May 2023). This includes addressing engineering concerns regarding heating and lightning attraction of black blades (Stehouwer et al. 2022), aviation regulations for either red tipped blades or red tower bands (Bos et al. 2024), and concerns of visibility perceptions (Höfer 2023). As yet, wind turbine manufacturers do not supply blades painted black, and painting them after installation results in the loss of the turbine warranty. Further technical improvements to blade patterning designs including alternative blade colour configurations (e.g., red) will therefore be important to investigate (Blary et al. 2026; Brighton et al. 2026; Hancock et al. 2026). Another priority action will the further implementation and monitoring of this measure at other sites across the globe to provide a stronger evidence base for its efficacy (Gottlieb et al. 2025; May 2023).

## 5 Conclusion

Following up on an earlier study in Norway, we could corroborate their findings on the efficacy of blade patterning in reducing bird collision fatalities. At the El Ruedo wind farm, bird fatality rates dropped by 74%, especially for passerines. The measure was also proven effective for large waterbirds, but less for birds of prey. This mitigation measure has now been shown to be functional at sites spanning a wide latitudinal range. Still, overcoming implementation hurdles and evaluating other site‐ and species‐specific conditions (e.g., offshore at Ecowende, onshore in Wyoming (USA)) require continued focus on additional testing, but not in the least in gaining more experience in practical implementation.

## Author Contributions

**Roel May:** formal analysis (lead), methodology (lead), visualization (lead), writing – original draft (lead), writing – review and editing (equal). **Miguel Ángel Hernández Gómez:** conceptualization (equal), data curation (equal), formal analysis (supporting), methodology (supporting), visualization (supporting), writing – original draft (supporting), writing – review and editing (equal). **Juan Luis Aguirre Martínez:** conceptualization (equal), data curation (equal), formal analysis (supporting), methodology (supporting), visualization (supporting), writing – original draft (supporting), writing – review and editing (equal). **Jenifer Andreu Miguel:** conceptualization (lead), data curation (equal), funding acquisition (lead), project administration (lead), writing – review and editing (equal).

## Conflicts of Interest

The authors declare no conflicts of interest.

## Data Availability

The R scripts and data file needed to perform all analyses are supplied as Supporting Information at the Mendeley Data repository (https://doi.org/10.17632/35zrd83gjp.1).

## Appendix 1 The Number of Bird Fatalities Recorded in the El Ruedo Wind Farm (Tarifa, Spain) in the Period 2012–2024

**Table:**

Species name	English name	Total number of fatalities	Control		Impact	
			Before	After	Before	After
**Passerines**		**106**	**59**	**22**	**23**	**2**
*Acrocephalus scirpaceus*	Eurasian reed warbler	1	1	0	0	0
*Anthus pratensis*	Meadow pipit	1	0	1	0	0
*Carduelis carduelis*	European goldfinch	6	4	0	2	0
*Cisticola juncidis*	Zitting cisticola	1	1	0	0	0
*Emberiza calandra*	Corn bunting	55	31	8	15	1
*Galerida cristata*	Crested lark	12	8	3	1	0
*Linaria cannabina*	Common linnet	3	0	1	2	0
*Melanocorypha calandra*	Calandra lark	3	1	1	1	0
*Passer domesticus*	House sparrow	18	10	6	1	1
*Passer hispaniolensis*	Spanish sparrow	5	2	2	1	0
*Sylvia atricapilla*	Eurasian blackcap	1	1	0	0	0
**Aerial insectivores**		**22**	**16**	**2**	**4**	**0**
*Apus apus*	Common swift	11	6	2	3	0
*Apus pallidus*	Pallid swift	3	3	0	0	0
*Delichon urbicum*	Western house martin	5	4	0	1	0
*Hirundo rustica*	Barn swallow	1	1	0	0	0
*Ptyonoprogne rupestris*	Eurasian crag martin	1	1	0	0	0
*Tachymarptis melba*	Alpine swift	1	1	0	0	0
**Birds of prey**		**80**	**51**	**9**	**17**	**3**
*Accipiter nisus*	Eurasian sparrowhawk	2	1	0	1	0
*Athene noctua*	Little owl	1	0	0	1	0
*Buteo buteo*	Common buzzard	3	1	0	2	0
*Circaetus gallicus*	Short‐toed snake eagle	5	3	0	1	1
*Circus aeruginosus*	Western marsh harrier	3	3	0	0	0
*Circus pygargus*	Montagu's harrier	1	1	0	0	0
*Elanus caeruleus*	Black‐winged kite	3	2	1	0	0
*Falco naumanni*	Lesser kestrel	2	2	0	0	0
*Falco tinnunculus*	Common kestrel	17	9	3	3	2
*Gyps fulvus*	Eurasian griffon vulture	18	12	2	4	0
*Hieraaetus pennatus*	Booted eagle	10	6	1	3	0
*Milvus migrans*	Black kite	10	7	1	2	0
*Tyto alba*	Barn owl	5	4	1	0	0
**Large waterbirds**		**72**	**44**	**7**	**21**	**0**
*Bubulcus ibis*	Cattle egret	41	29	4	8	0
*Ciconia ciconia*	White stork	25	12	3	10	0
*Egretta garzetta*	Little egret	4	2	0	2	0
*Nycticorax nycticorax*	Black‐crowned night heron	1	0	0	1	0
*Platalea leucorodia*	Eurasian spoonbill	1	1	0	0	0
**Other bird species**		**17**	**8**	**4**	**5**	**0**
*Alectoris rufa*	Red‐legged partridge	4	2	0	2	0
*Anas platyrhynchos*	Mallard	3	1	0	2	0
*Coloeus monedula*	Eurasian jackdaw	1	0	1	0	0
*Columba livia*	Rock dove	2	2	0	0	0
*Larus fuscus*	Lesser black‐backed gull	1	1	0	0	0
*Merops apiaster*	European bee‐eater	2	1	1	0	0
*Phalacrocorax carbo*	Great cormorant	2	0	1	1	0
*Streptopelia decaocto*	Eurasian collared dove	1	1	0	0	0
*Vanellus vanellus*	Northern lapwing	1	0	1	0	0
